# Bimodal Control of Heat Transport at Graphene–Metal Interfaces Using Disorder in Graphene

**DOI:** 10.1038/srep34428

**Published:** 2016-10-04

**Authors:** Jaehyeon Kim, Muhammad Ejaz Khan, Jae-Hyeon Ko, Jong Hun Kim, Eui-Sup Lee, Joonki Suh, Junqiao Wu, Yong-Hyun Kim, Jeong Young Park, Ho-Ki Lyeo

**Affiliations:** 1 Graduate School of EEWS, Korea Advanced Institute of Science and Technology (KAIST), Daejeon 305–701, Republic of Korea; 2 Center for Nanomaterials and Chemical Reactions, Institute for Basic Science (IBS), Daejeon 305–701, Republic of Korea; 3 Korea Research Institute of Standards and Science (KRISS), Daejeon 305–340, Republic of Korea; 4 Graduate School of Nanoscience and Technology, Korea Advanced Institute of Science and Technology (KAIST), Daejeon 305–701, Republic of Korea; 5 Department of Materials Science and Engineering, University of California at Berkeley, Berkeley, California 94720, USA

## Abstract

Thermal energy transport across the interfaces of physically and chemically modified graphene with two metals, Al and Cu, was investigated by measuring thermal conductance using the time-domain thermoreflectance method. Graphene was processed using a He^2+^ ion-beam with a Gaussian distribution or by exposure to ultraviolet/O_3_, which generates structural or chemical disorder, respectively. Hereby, we could monitor changes in the thermal conductance in response to varying degrees of disorder. We find that the measured conductance increases as the density of the physical disorder increases, but undergoes an abrupt modulation with increasing degrees of chemical modification, which decreases at first and then increases considerably. Moreover, we find that the conductance varies inverse proportionally to the average distance between the structural defects in the graphene, implying a strong in-plane influence of phonon kinetics on interfacial heat flow. We attribute the bimodal results to an interplay between the distinct effects on graphene’s vibrational modes exerted by graphene modification and by the scattering of modes.

Amongst the remarkable properties of graphene (e.g., electronic^1^,^2^,^3^, mechanical^4^, and thermal^5^,^6^,^7^,^8^,^9^) is its extraordinary capability to carry heat (i.e., thermal conductivity) at a rate higher than 4000 W m^−1^ K^−1^ at ambient temperature.^5^ Advent of this two-dimensional material consisting of a single-atom-thick network of carbon hexagons has spurred a great deal of research on low-dimensional heat conduction^7^,^8^. A collective vibration of carbon atoms in the graphene lattice is mostly responsible for the transport of thermal energy^5^,^7^,^8^. Three particular polarizations of acoustic vibrational modes are involved in thermal transport: two in-plane modes (i.e., longitudinal and transverse modes) and one out-of-plane mode (i.e., flexural mode)^8^,^9^. Heat transfer between graphene and its surroundings occurs along the direction of the reduced dimension in the graphene. Such conduction of heat is known to be governed by the transmission of vibrational modes (or phonons) across the interface, which depends on the elastic properties of the materials involved^7^,^8^,^9^,^10^,^11^. This characteristic of thermal transport also dominates heat flow across the graphene–metal interface, which is largely limited by the elastic properties of the metal because of limited coupling with phonons in the pristine graphene^8^,^12^,^13^,^14^,^15^,^16^.

To achieve an appreciable control of heat flow in a system involving graphene, one needs to consider modifying the material at the microscopic length scale. In this regard, chemical adsorption on the graphene surface^15^, strain engineering^17^, and defect engineering^18^ were suggested recently, which would accompany the changes in the elastic properties of graphene down to atomic scale. In spite of the experimental and theoretical efforts, it is yet unclear how microscopic changes are associated with thermal conduction across the interface.

In this paper, we show how the vibrational spectra in graphene change with different types of disorder and how such a change can be related to heat transfer between graphene and metals, where the graphene was modified in an attempt to manipulate the phonons and thus thermal transport at the interface. We traced the development of thermal transport in response to increasingly modified phonons, which reveals very different effects of disorder on heat transport across the graphene–metal interfaces. Elucidating this relation can strengthen the possibilities for controlling heat transport by exploiting the microscopic and low-dimensional heat conduction pathways.

## Results

### Producing physical and chemical defects in graphene

**Figure 1:**
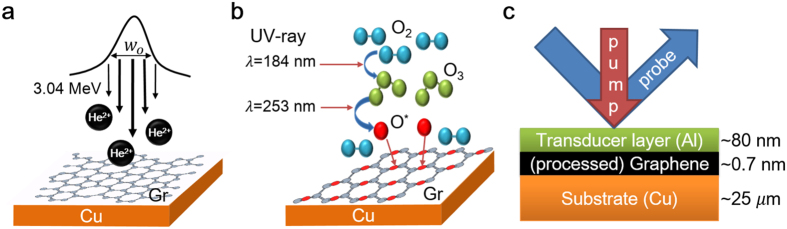
Illustrations showing modification of the graphene and sample structures for the TDTR measurements. (**a**) Ion-beam irradiation with a Gaussian distribution. The 1/*e*^2^ diameter *w*_*o*_ of the beam intensity was 6.4 mm and the peak ion dose was ~10^15^ cm^−2^ at a kinetic energy of 3.04 MeV. (**b**) A UV lamp with two mixed wavelengths was used to produce oxygen radicals and induce the chemical reaction with graphene (see Methods). (**c**) Cross-sectional view of the sample structure for thermal conductance measurements. Following the processes illustrated in Fig. 1a,b, we deposited an Al transducer layer with an optically opaque thickness of ~80 nm to perform the TDTR experiments. All measurements were performed at room temperature.

To investigate how interfacial heat flow responds to the disorder in graphene, we modified graphene by generating either physical or chemical disorder. Different types of disorder were produced in the graphene through two separate processes: ion bombardment and oxidation. Irradiation using a He^2+^ ion beam with a Gaussian distribution creates a spatial distribution of structural defects in the graphene lattice, as depicted in Fig. 1a. On the other hand, varying exposure to an ultraviolet (UV)–ozone environment induces oxidation of the graphene, which is a previously known process^19^,^20^. This method exerts a series of chemical modifications on the graphene lattice, as illustrated in Fig. 1b. We primarily employed Raman spectroscopy to characterize the amount of disorder before and after the processes. Following the spectral measurements of the Raman signal, we deposited an Al film on the graphene using DC-magnetron sputtering to take time-domain thermoreflectance (TDTR) measurements. The optically opaque Al film with a thickness of ~80 nm serves both as a metal contact forming a junction with the graphene and as a transducer layer for the TDTR method^12^,^21^,^22^, which enables measurement of the thermal conductance across the metal–graphene junction (see Fig. 1c).

### Influence of physical defects on thermal transport at graphene-metal interfaces

**Figure 2:**
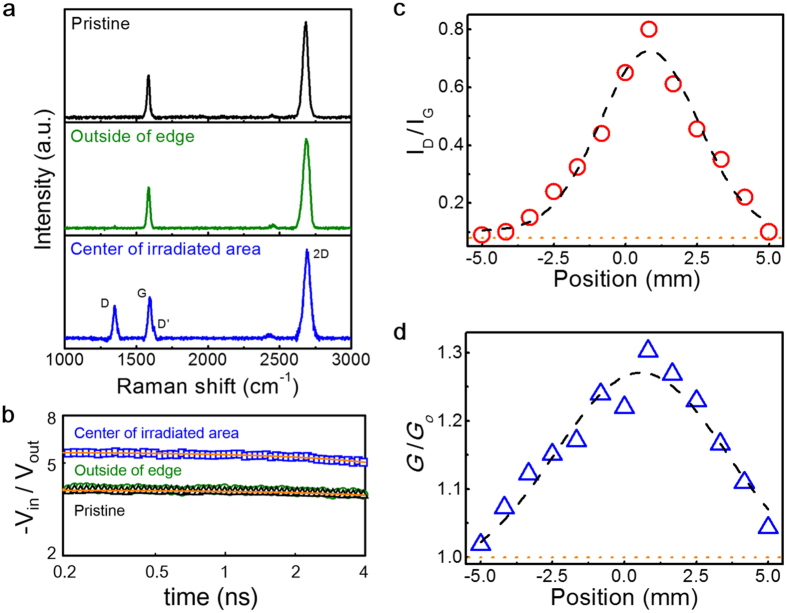
Profiling measurements of the Raman spectra and thermal conductance at room temperature. (**a**) Raman spectra obtained from pristine graphene (top) and ion-bombarded graphene (center and bottom). The center and bottom spectra were taken at locations outside and at the center of the ion-irradiated area, respectively. (**b**) TDTR data are shown and used to extract the thermal conductance *G* for the Al/graphene/Cu junction. Conductance values measured at the center, outside the edge, and on pristine graphene are 28.6, 21.8, and 21.3 MW m^−2^ K^−1^, respectively. To extract the conductance values, we used the measured thermal conductivity of the Al film and Cu substrate (see Methods). The thermal conductivity of the Cu substrate changes with ion irradiation (see Supplementary Information). Repeated measurements yielded slightly different values. (**c**) Raman spectra were obtained across the diameter of the ion-beam irradiation. The plot shows the ratio of the ‘D’-to-‘G’ peak intensity along the diameter. (**d**) The change in thermal conductance relative to the case of pristine graphene is shown along the same diameter as in Fig. 2c. The error bar is not plotted because the uncertainty of the repeated measurements is smaller than the symbol size. The dashed lines in c and d are Gaussian fittings along the irradiated beam spot. The seemingly asymmetric distribution is because the irradiation occurred closer to one edge of the sample.

Signatures of the structural changes made in the lattice by ion irradiation and its influence on thermal transport can be readily seen in Fig. 2a,b with respect to pristine graphene. Raman spectra obtained outside of the irradiating beam resemble the pristine case, as shown in Fig. 2a. Both spectra exhibit the typical responses of Raman measurements of pristine graphene and only a tiny signature indicating defects. In contrast, the spectra obtained near the center of the ion beam indicate the presence of a considerable number of defects (i.e., the defect signature rises clearly at ~1350 cm^−1^)^23^. Figure 2b shows a notable difference between the temporal responses from the TDTR measurements, implying that these defects produced by the ion bombardment significantly affect heat transfer across the graphene junction. The thermal conductance measured at the edge is similar to the pristine case, as expected, but the conductance measured at the center increases substantially (by ~30%), which was extracted from the TDTR data displayed in Fig. 2b. The thermal conductance^10^ *G* is defined as a coefficient of heat flow Q across the graphene junction by considering the thickness of graphene (0.35 nm) and volumetric heat capacity (0.767 J cm^−3^), where *Q* *=* *G ∆T* with an abrupt temperature drop *∆T* at the junction.

The temporal profiles of the ratio of the in-phase and out-of-phase signals *−*V_in_/V_out_, shown in Fig. 2b, represent how optical reflectivity changes with temperature in the probed area (~26 *μ*m^2^) in response to modulated heating due to the pumping laser beam (see Methods). Analysis of the probe–beam response obtained from the TDTR measurements yields a thermal conductance of *G* ≈ 21 MW m^−2^ K^−1^ for the junction containing pristine graphene grown on Cu foil. For pristine graphene grown on a different Cu foil, the measured conductance increases by ~50% (i.e., *G* ≈ 31 MW m^−2^ K^−1^) as shown in Supplementary Fig. S2a. We attribute this conductance change to a different contact area at both sides of the graphene interface, which is rather in accordance with previous studies^24^,^25^ (see Supplementary Fig. S2). Despite the change, the measured value is similar to the previously reported conductance of 20 < *G* < 30 MW m^−2^ K^−1^ for junctions such as Au/Ti/graphene/SiO_2_ and Al/graphene/SiO_2_ at room temperature^12^,^15^.

Thermal transport across the Al/graphene/Cu junction is mediated by monolayer graphene sandwiched between two different metals, where the graphene is processed to contain a varying amount of structural or chemical disorder. In the sandwich structure, the thermal resistance 1/*G* of the junction could be expressed as a sum-of-resistance series


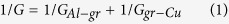


where *G*_*Al-gr*_ and *G*_*gr-Cu*_ are the thermal conductances of the Al–graphene and graphene–Cu interfaces, respectively. The internal thermal resistance per unit area of graphene can be regarded to be small compared with the resistance at the interfaces, which is ~10^−8^ m^2^ K W^−1^, because the internal resistance would be, at most, on the order of 10^−9^ m^2^ K W^−1 12^. We may therefore assume that interfacial heat transport mostly determines the total resistance 1/*G* across the graphene–metal junction and any change that may result from modification of the graphene lattice. We discuss below how thermal transport evolves with disorder in the graphene and what causes the change.

### Spatial profiles of defect density and thermal conductance

To investigate the influence of structural disorder on thermal transport, we produced spatially varied defects in the graphene and carried out profiling measurements using both Raman spectra and thermal conductance along the bombarding ion beam. Spatial distribution of the structural disorder was produced in graphene by irradiation using a He^2+^ ion beam with a Gaussian distribution. The 1/*e*^2^ radius of the beam intensity was 3.2 mm and the peak ion dose was ~10^15^ cm^−2^ at a kinetic energy of 3.04 MeV. We then traced the defect signature using Raman spectroscopy. Figure 2c shows the profile of the ratio between the ‘D’ band intensity at wavenumber ~1350 cm^−1^ and the ‘G’ band intensity at ~1580 cm^−1^, as obtained from the Raman measurements. The profile clearly follows a Gaussian distribution and 1/*e*^*2*^ distance of the distribution approximately matches the diameter of the ion beam. The seemingly asymmetric distribution is because the irradiation occurred closer to one edge of the sample. These results suggest that the density of the produced defects could be proportional to the bombarding He^2+^ beam intensity.

Analysis of the ‘D’ band intensity I_D_ and ‘G’ band intensity I_G_ allows us to estimate the defect density. With the peak dose roughly equivalent to 1 × 10^15^ He^2+^ cm^−2^, we find that the defect density falls to a low-defect density regime^26^ in which the average distance between defects L_D_ is greater than 10 nm. This contrasts with Ar^+^ ion bombardment at a similar dose, which generates a much larger number of defects^26^. The discrepancy is primarily because the probability of creating a defect is negligibly small when using He^2+^ compared with using heavier but lower energy ions (e.g., Ne^+^ and Ar^+^)^23^. In the low-density regime, the intensity I_D_ is simply proportional to the total number of defects probed by the Raman laser spot because the Raman signal becomes an independent sum of the contributions from each defect^26^,^27^. The ratio of ‘D’ to ‘G’ band intensity (i.e., I_D_/I_G_) can then be related to the average inter-defect distance L_D_ by an empirical formula^26^ 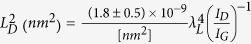 where λ_L_ is the wave length of the Raman laser beam at 514 nm. This leads to a rather simple relation 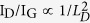, which implies that the ratio is proportional to the defect density. The distance L_D_ at the center position and edge of the beam profile can be estimated as 13.2 ± 3.6 nm and 34.5 ± 9.6 nm, respectively. Hence the defect density should be less than 0.1% even at the center position.

The measured thermal conductance also changes with ion beam intensity, as shown in Fig. 2d. The profile of the change in conductance can also be fitted to a Gaussian distribution, but with a larger width than the defect signature in the Raman measurements. In the TDTR analysis of the irradiated sample, a notable result is that the thermal conductance increased by as much as ~30%, which is a substantial change compared with the relatively small modification of its structure.

To gain insight into how the defects affect heat transport, we examine a correlation between the measured defect distribution and thermal conductance. Since both the profiles of I_D_/I_G_ and *G*/*G*_*o*_ follow Gaussian distributions, the use of a proportional relation between L_D_^−2^ and I_D_/I_G_ allows us to extract a relation between L_D_ and the thermal conductance. A Gaussian distribution can be expressed as 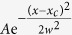 where *w* is the distribution width, *x* and *x*_*c*_ are the measurement location and peak position of the distribution, respectively, and *A* is a constant. Assuming a dependence of thermal conductance on the distance L_D_ as 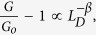, we can extract that the exponent should be 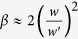, where *w* is the width from the defect density distribution and *w*′ is the width of the Gaussian distribution fitted to *G*/*G*_*o*_ − 1. From the data in Fig. 2d, we obtain β ≈ 0.9 ± 0.1, which implies that the increase in conductance is approximately inverse-proportional to the distance L_D_, whereas β ≈ 2 holds quite well for the Raman ‘D’ band profile (i.e., I_D_/I_G_).

This result is evidence that a kinetic in-plane interaction between the defects strongly influences the cross-plane thermal transport occurring at the interface. The kinetic description of thermal conductivity Λ∼*Cvl* states that the phonon mean-free-path *l* is longer than 100 nm in graphene, where *C* is the specific heat capacity and *v* is the speed of sound^28^. The phonon mean free path (MFP) in graphene is the sum of two MFPs limited by Umklapp scattering (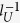) and point-defect scattering (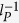) as 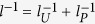
6. If a kinetic process is dominated by point-defect scattering, a change in *l*_*P*_ will determine the total MFP change and thus thermal conductivity. While the shortened distance between defects and accordingly reduced MFP should significantly impair in-plane heat transport^9^, kinetic considerations suggest that interfacial transport is affected by in-plane scattering of the phonons. An important question then arises: why is cross-plane thermal transport enhanced when phonons are more frequently scattered along in-plane? Before discussing this matter, we first explore a distinct case.

### Changes in thermal conductance with varying degrees of chemical modification

**Figure 3:**
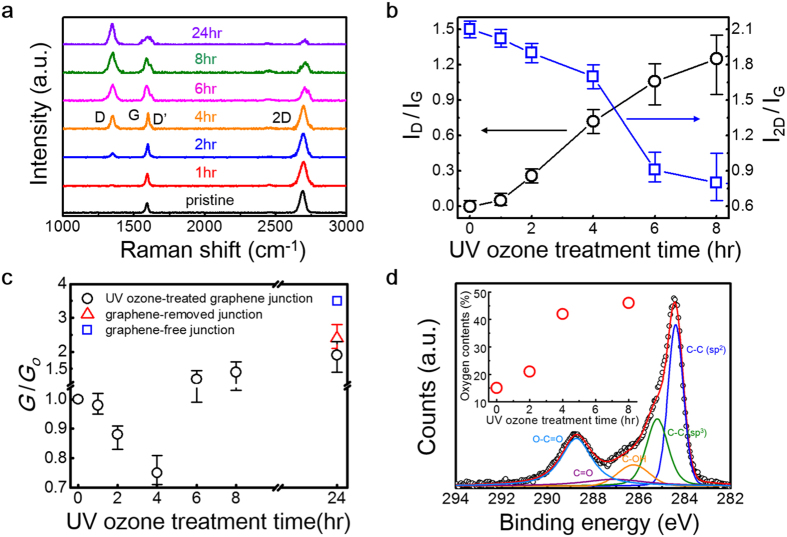
Development of Raman spectra, X-ray photoemission spectroscopy, and thermal conductance in response to the UV–ozone process. (**a**) Evolution of Raman spectra obtained from graphene processed with increasing exposure to UV–ozone. (**b**) Plot of the development of Raman intensities of the ‘D’ (black circle) and ‘2D’ (blue square) bands with respect to the ‘G’ band with varied exposure times. The error bar was determined from repeated measurements at different locations. The data for 24-hour treatment is not shown here because of large fluctuations dependent on the measurement location. The connecting lines are only for guiding the eye. (**c**) Thermal conductance change with increasing exposure is plotted along with the values measured for the graphene-removed (red triangle) and graphene-free (blue square) samples. The error bar represents both the uncertainty of repeated measurements and spatial non-uniformity. Note the breaks on the x- and y-axes; the scales are linear before and after the breaks. (**d**) Spectra of binding energy obtained from XPS measurements of graphene processed for 4 hr. The plot in the inset indicates the changes in the portion of carbon–oxygen bonds with respect to carbon–carbon bonds. The XPS measurement of UV–ozone-treated graphene shows a broadening of the C1s peak because of the emergence of sp^3^ C–C bonds (285.1 eV), which indicates the break-up of sp^2^ C=C bonds (284.4 eV) in the graphene. The deconvoluted spectra reveal the formation of various chemical groups involving the carbon and oxygen atoms (e.g., C–OH (~286.5 eV), C=C–OH (~288.6 eV), and O–C=O (~289.2 eV))^19^,^20^. Both the spectra and oxygen content are similar to previous results that used a similar treatment method^19^,^20^.

For comparison, we investigate how heat flow responds to chemical modification of the graphene lattice by adsorbed foreign species. Graphene was processed by varying its exposure to a UV–ozone environment. This method utilizes UV rays to induce the chemical reaction of oxygen radical molecules with the graphene surface by generating ozone molecules from UV-activated oxygen (see Methods for details)^19^,^20^. Subsequent Raman measurements reflect varying oxidation of the graphene, as shown Fig. 3a. As we show in Fig. 3b, the intensities of the ‘D’ and ‘D′’ bands increase with increasing exposure, whereas the intensity of the ‘2D’ band decreases relative to the ‘G’ band. The observation that the ratio I_D_/I_G_ increases approximately linearly with exposure time after the initial exposure suggests an increase in oxidation coverage proportional to the processing time.

The development of the Raman bands is similar to that of ion-bombarded graphene, but the trend in thermal conductance appears largely different and even opposite. In Fig. 3c, we show the changes in thermal conductance due to chemical processes. In the relatively early stages (i.e., up until four hours of treatment), the measured conductance is rather uniform over the sample and declines roughly linearly by as much as ~30%. With further processing, however, thermal conductance abruptly rises by ~100%, as shown in Fig. 3c, which is ~50% higher than that of a pristine graphene junction. We note that the relatively large uncertainty of the measured conductance is a result of spatial non uniformity over the sample surface. Upon a longer processing period (i.e., for 24 hours), the conductance continues to increase. In contrast to the large modulation in conductance, the trend in the Raman signals remains unchanged, as shown in Fig. 3b, which implies a continuation of the oxidation process. Measurement of the chemical groups using X-ray photoemission spectroscopy (XPS) confirms the increase of carbon–oxygen bonds, as displayed in Fig. 3d and the inset. These results show both a similarity and discrepancy compared with previous work^15^ that reported a similar increase in conductance for an Al/graphene/SiO_2_ junction as a result of oxidation using oxygen plasma, in which the enhanced conductance was attributed to an increased strength of the chemical bonding between the Al layer and the oxygen-adsorbed graphene. Despite the similar results, the reduced conductance observed in the earlier stages of oxidation, shown in Fig. 3c, cannot be accounted for by bond strength.

To appreciate the abrupt rise in thermal conductance and the measurement uncertainty following longer graphene processing times (over 6-hour), we measure the conductance of graphene-removed and graphene-free junctions. The graphene was intentionally removed using oxygen plasma to prepare the graphene-removed junction. The graphene-free junction was prepared by simply depositing an Al thin film on a Cu substrate. Measured values for both samples are plotted in Fig. 3c along with the 24-hour graphene processing result. In the graphene-free junction, the measured conductance of *G*_*Al-CuO/Cu*_ ~100 MW m^−2^ K^−1^ is higher than that of the graphene-removed junction, but it is significantly lower than *G*_*Al-Cu*_ ~4 GW m^−2^ K^−1^ for the Al–Cu interface^29^ because of inactive electronic contributions to thermal conduction from the formation of native copper oxide (CuO). Note that the conductance obtained from the increasingly oxidized samples approaches the value measured on the graphene-removed sample. In addition to the observation of the large spatial non-uniformity in both the conductance and Raman signatures from the longer-processed graphene, the roughly asymptotic behavior with increasing oxidation suggests thermal conduction through direct contact between the deposited Al film and the CuO/Cu substrate. Supposing that a fraction of the graphene is removed by the UV–ozone process, heat will flow in parallel across the junction. We can thus write the junction conductance *G′* as


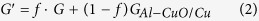


where *f* is the areal fraction of the remaining graphene and equation (1) defines *G*. This relation gives us simple estimates of the fraction (i.e., *f* > 95%, *f* > 90% and *f* ~75% for the 6-hour, 8-hour and 24-hour cases, respectively), implying that the conductance may vary considerably with a relatively small fraction of direct contact. The abruptly large change in the conductance can thus be accounted for by partial removal of the graphene after broad coverage of carbon–oxygen bonds. Indeed, as shown in the inset of Fig. 3d, only a slight increase in carbon–oxygen content during the period also supports the explanation.

## Discussion

**Figure 4:**
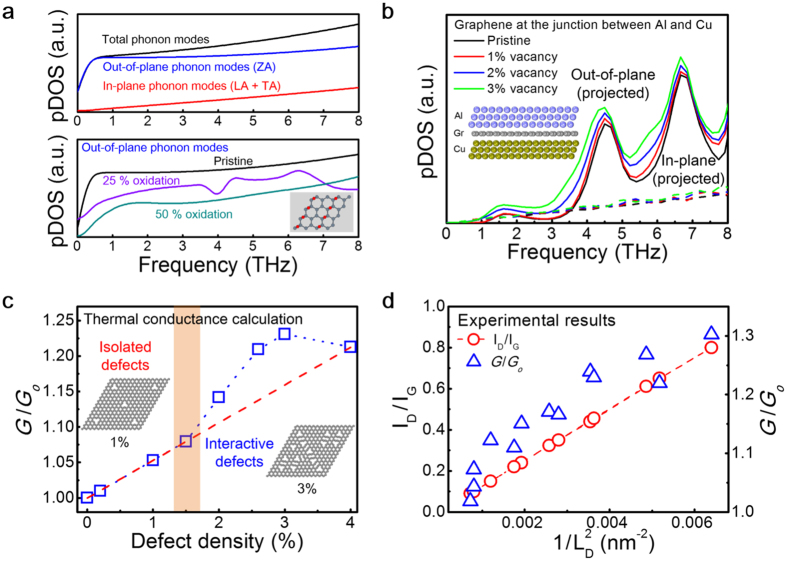
Comparison of experimental results and calculated results for pDOS and thermal conductance. (**a**) Calculated pDOS for pristine and oxidized graphene. The top graph exhibits the pDOS calculated for the pristine graphene. The bottom graph shows the pDOS of the out-of-plane phonon modes calculated for the three cases of oxygen content (i.e., pristine, 25%, and 50%) below 8 THz. Upon oxidation, the pDOS of the low-frequency flexural phonon mode is reduced because of the oxygen adsorbates. (**b**) Plot of the calculated pDOS projected on each direction for the pristine and defective graphene sandwiched between Al and Cu, as illustrated in the inset. The increase in the out-of-plane pDOS is more notable than in the other direction. (**c**) Computed change in thermal conductance with increasing defect density. The shaded area implies a transition between isolated defects and kinetically interactive defects through the Tersoff potential (see Supplementary Information). The vacancies incorporated into the graphene layer are shown with 1% and 3% defect densities. The red dashed line indicates the change in conductance with the assumption of isolated defects. (**d**) Experimental ratio of Raman intensity and thermal conductance plotted with defect density 1/L_D_^2^. While the Raman signal ratio (red circle) is directly proportional to 1/L_D_^2^, the measured thermal conductance (blue triangle) deviates from linear proportionality to defect density (i.e., increases with 1/L_D_).

On the other hand, the reduction in thermal conductance observed in the earlier stages of UV–ozone processing is distinguished from previous results and remains to be explained. Since the elastic property of graphene changes with oxidation^30^,^31^, we speculate that the decline in conductance is caused by modification of the vibrational modes in the graphene by the adsorbed species. This is because the vibrational (i.e., phonon) density of states (pDOS) of oxygen-adsorbed graphene tends to be reduced from the pDOS of pristine graphene at relatively low frequencies (i.e., ~8 THz), as computed and shown in Fig. 4a. Such a reduction appears particularly notable below the cut-off frequency of the adjoining metal’s phonons, which can contribute to coupling with the phonons of graphene; the cut-off frequencies of the longitudinal (transverse) vibrational modes for Al and Cu are ~9 (5) THz and ~8 (5) THz, respectively^32^,^33^. Assuming diffusely scattered phonons at the graphene–metal interface, the lower cut-off frequencies of the metals and reduced pDOS of the oxidized graphene are expected to limit thermal transport across the graphene junction. As a result, with the assumptions of the diffuse mismatch model (DMM), the phonon transmission probability across the junction is reduced and the conductance decreases, too (see Supplementary Information for the details). This picture is consistent with the change in mechanical properties of the graphene oxide (i.e., in-plane stiffness and bending stiffness). Formation of carbon–oxygen bonds lowers the Young’s modulus of the graphene membrane^30^,^31^, which is a measure of in-plane stiffness. By contrast, the adsorbed molecules tend to increase the bending stiffness in graphene along the out-of-plane direction, as demonstrated in the friction measurements of fluorinated graphene^34^, which was attributed to a similar change in pDOS as in the present thermal transport case.

There are two more factors to consider regarding the oxidizing method and the material system. XPS measurements indicate that the signature of the carbon–oxygen bonds and a portion of the functional group with a higher bonding energy (i.e., O-C=O) are larger in our method than in the previous report^15^ using oxygen plasma, as shown in Fig. 3d. Such a prominent existence of functional group might be a characteristic of UV–ozone treatment because our results are quite similar to previous results^19^,^20^ that used the same method. The substrate is different, too, where the thermal conductance between Al and SiO_2_ is known to be ~200 MW m^−2^ K^−1^, which is a factor of two greater than *G*_*Al-CuO/Cu*_. Yet these factors are insufficient when explaining the seemingly discrepant conclusions because the thermal transport pathway is likely to be similar. Although we exhibit the varying stages in thermal conductance in response to a range of oxidation, the cause of the discrepancy remains unclear.

We address the distinct behavior of thermal conductance obtained from ion-irradiated graphene in which the conductance increased with the creation of more defects. First, we consider a few experimental factors that may result in such an increase in conductance. (1) The thermal conductivity of the Cu substrate (Λ_*Cu*_) could change after ion irradiation and affect thermal transport across the junction. (2) We consider the possibility of forming a direct contact between the Al film and substrate through graphene pinholes that might be created by the ion bombardment, which would result in larger conductance. Bombarding the graphene on Cu substrate not only produces defects in the graphene, but also damages the substrate. Indeed, the expected damage reduced the thermal conductivity Λ_*Cu*_ by ~10% (see Supplementary Fig. S1). However, the thermal conductance across the junction is relatively insensitive to the change (i.e., the conductance varies by only a couple of percent even with a 50% change in Λ_*Cu*_). Additionally, the supposed thermal transport through pinholes would contribute to thermal conductance, as included in equation (2). For instance, a coverage of 0.1% pinholes would contribute to an increase in the conductance of less than 0.1 MW m^−2^ K^−1^, which is negligible compared with the observed enhancement. Furthermore, the grain size of the Al thin film is a few tens of nanometers (Supplementary Fig. S3), which is much larger than the supposed atomic-scale graphene pinholes. We can therefore regard the contribution of these factors to thermal conductance as insignificant.

Having considered the experimental factors, we now examine the influence of ion-irradiation on the vibrational eigenmodes of the graphene lattice and heat flow by means of phonon transport. Irradiating graphene with energetic He^2+^ ions produces physical defects and breaks the symmetry of the graphene lattice. Breaking the three-fold symmetry causes the emergence of mixed eigenmodes of pristine graphene near the defect sites, which appears to be an oblique motion of atoms (see Supplementary Information and Supplementary video SV1). We computed the vacancy-induced modifications in the vibrational modes of graphene and their transmission across the interface. Upon calculating the vibrational modes of the defective graphene sandwiched between the Al and the Cu substrate, the projected pDOS of each polarization is displayed in Fig. 4b. An increase in pDOS due to vacancies can be seen in particular from the out-of-plane modes. As a result, the thermal conductance across the junction increases with increasing defect density, which is shown in Fig. 4c. We note that the out-of-plane modes are dampened by being shifted toward higher frequencies due to the substrate. More importantly, despite the damping, thermal transport across the junction is governed by the transmission of out-of-plane vibrational modes rather than in-plane modes (see Supplementary Information). This calculation again validates the prior argument that the reduced thermal conductance observed in the earlier stages of oxidation is mostly due to a decrease in the out-of-plane phonon modes causing reduced pDOS and conductance. These results are in accordance with theoretical work that predicted enhanced thermal conductance between graphene and MoS_2_ with an increased vacancy density in graphene^18^.

Computational simulation also helps to account for the influence of inter-defect interactions on thermal conductance. The shaded region near ~1.5% in Fig. 4c represents a transition between the influence of isolated defects and interactive defects on thermal transport, which reflects the range of the Tersoff inter-atomic potential used for simulating the graphene layer (see Supplementary Information). The calculated conductance increases linearly with defect density in the non-interactive regime because of the independent contribution of increased pDOS owing to each defect. The conductance deviates from a linear dependence as the inter-defect distance becomes comparable to or smaller than the range of the model potential, which will involve in-plane scattering of phonons. We attribute this effect to the induced vibration of atoms neighboring point defects within the model potential (see Supplementary Information and Supplementary Video SV1), which can contribute to additional pDOS. This process resembles the reduced in-plane phonon MFP with increasing defect density as shown in the profiling measurements. In fact, experimental results exhibit such deviation from the assumption of isolated defects because of the kinetic interaction between defects within the MFP, as shown in Fig. 4d. These results suggest that the contribution of kinetically interactive defects is a likely pathway for both the enhancement and spatial change of thermal conductance observed in the experiments. This effect can now be readily appreciated; at the experimental defect density of ~0.1%, the enhancement in conductance reaches ~30% but the isolated contribution of each defect is estimated to be only ~1% or less as shown in Fig. 4c.

We note that the simulated results significantly underestimate the conductance. This is because the extent of the inter-atomic potential excludes the contribution of farther defects. On the other hand, the defect density used for the computation was largely exaggerated because of the short range of the potential and size limitation in modelling. The practical limit in the model can be seen in Fig. 4c, where the declining conductance at densities over 3% is caused by weakened coupling of the distorted graphene with neighboring materials.

In summary, we showed how structural disorder in graphene exerts distinct changes on the vibrational states of graphene and, eventually, thermal transport across the Al/graphene/Cu junction. Physical defects produced by ion irradiation cause increased thermal conductance across the graphene–metal interface. That is because the creation of physical defects in graphene triggers two inter-related effects on cross-plane thermal transport across junction interfaces: additional vibrational states leading to enhanced pDOS and the scattering of phonons limiting heat flow that appear as a kinetic dependency on the inter-defect distance. In contrast, we observed a large modulation in thermal conductance as chemical modification of the graphene developed via oxygen functionalization. This phenomenon was attributed to a change in the out-of-plane elastic properties that were affected by the reduction in pDOS as well as partial removal of graphene at relatively high oxygen coverage. The distinct effects, depending on the modification of the graphene, were nearly indistinguishable in the Raman spectra, but appeared as a clear difference in the thermal transport. We believe these results can have implications in controlling the thermal transport at graphene–metal junctions that are technologically important for devising future devices through microscopic changes in the elastic properties of graphene.

## Methods

### Sample and Raman measurement

We purchased commercial graphene samples that were grown on 25-μm-thick Cu foil using the chemical vapor deposition method. We also purchased Cu-foil substrates, graphene grown on a Cu foil with different morphologies, and graphene-removed Cu. We used a LabRAM HR UV-VIS-NIR Raman microscope (Horiba Jobin Yvon) to obtain Raman spectra of the graphene. The laser wavelength for Raman excitement and beam spot size were 514 nm and ~3 μm^2^, respectively, at ambient conditions. To avoid sample heating, the power of the Raman laser beam was maintained at 0.5 mW.

### UV–ozone treatment

This process uses UV lights with wavelengths λ of 184 and 253 nm. Ozone molecules, which are generated by UV (λ = 184 nm) activation of oxygen, sequentially decompose to oxygen radicals that can oxidize graphene, which is assisted by UV rays at λ = 253 nm. These oxygen radicals are known to absorb on the graphene basal plane and chemically react with the carbon atoms to form functional groups (e.g., epoxide and carbonyl groups^19^,^20^). For quantitative analysis of the oxidation effect, the graphene surface was processed with ozone exposure for 1-, 2-, 4-, 6-hr and longer processing (up to 8 hr) at room temperature.

### TDTR

TDTR is an optical pump–probe technique that can measure the thermal conductivity Λ of thin films^21^,^22^ and the thermal conductance *G* between materials^35^. The method traces a temperature-dependent change in reflectivity that yields in-phase V_in_ and out-of-phase V_out_ signals in response to a modulated pump beam, where the 1/*e*^*2*^ radius of the probe beam ≈5.1 μm. We analyzed the ratio −V_in_/V_out_ to obtain *G* for the Al/graphene/Cu junction. An optically opaque Al film that is ~80 nm thick is necessary for the TDTR method we used to extract the thermal properties of graphene. Separate measurements of the thermal conductivity of the Al film and Cu substrate essentially leave the thermal conductance of the junction as the only unknown quantity in the analysis of the TDTR signal.

In thermal modeling, there is only one free parameter: the thermal conductance of the Al/Gr/Cu interfaces. The thermal conductivity of Al and Cu were obtained using the Wiedemann–Franz law (i.e., Λ = *L*σ*T* where *L* is the Lorentz number, σ is the electrical conductivity, and *T* is the absolute temperature). The Lorentz numbers for Al and Cu are slightly different because of the interaction of electrons with the lattice vibration^36^. We took into account both the thickness of the graphene (0.35 nm) and volumetric heat capacity (0.767 J cm^−3^).

## Additional Information

**How to cite this article**: Kim, J. *et al*. Bimodal Control of Heat Transport at Graphene–Metal Interfaces Using Disorder in Graphene. *Sci. Rep*. **6**, 34428; doi: 10.1038/srep34428 (2016).

## Supplementary Material

Supplementary Information

Supplementary Movie S1
