# Supplementary material for: Bimodal Control of Heat Transport at Graphene–Metal Interfaces Using Disorder in Graphene
Source: Sci Rep. 2016 Oct 4;6:34428. doi: 10.1038/srep34428 (PMC5048174; doi:10.1038/srep34428)
Supplement: Supplementary Information [file srep34428-s1.pdf]

Supplementary Information

Bimodal Control of Heat Transport at Graphene–Metal Interfaces using Disorder in Graphene

Jaehyeon Kim[1,2,3], Muhammad Ejaz Khan[4], Jae-Hyeon Ko[4], Jong Hun Kim[1,2], Eui-Sup Lee[4], Joonki Suh[5], Junqiao Wu[5], Yong-Hyun Kim[4*], Jeong Young Park[1,2 *], and Ho-Ki Lyeo[3*]

[1]Graduate School of EEWS, Korea Advanced Institute of Science and Technology (KAIST) Daejeon 305-701, Republic of Korea. [2]Center for Nanomaterials and Chemical Reactions, Institute for Basic Science (IBS), Daejeon 305-701, Republic of Korea. [3]Korea Research Institute of Standards and Science (KRISS), Daejeon 305-340, Republic of Korea. [4]Graduate School of Nanoscience and Technology, Korea Advanced Institute of Science and Technology (KAIST) Daejeon 305-701, Republic of Korea. [5]Department of Materials Science and Engineering, University of California at Berkeley, Berkeley, California 94720, USA.

*To whom correspondence should be addressed. E-mail: yong.hyun.kim@kaist.ac.kr, jeongypark@kaist.ac.kr, hklyeo@kriss.re.kr

Table of contents

S1. Influence of the thermal conductivity of Cu substrate on the measurement of thermal transport across graphene junction

S2. Influence of contact area on the thermal transport measurement

S3. Topographic morphology of the Al thin film

S4. Thermal conductance upon extended exposures to the UV–ozone environment

S5. Analysis of interfacial thermal conduction using the diffuse mismatch model

S6. Computational method: First-principles density functional theory calculation for pristine and oxidized graphene

S7. Computational method: The non-equilibrium Green's function approach for the interface between the structurally disordered graphene and a metal

S8. Flexural pDOS analysis to elucidate increasing thermal conduction with graphene/metal vibrational coupling

S1. Influence of the thermal conductivity of Cu substrate on the measurement of thermal transport across graphene junction

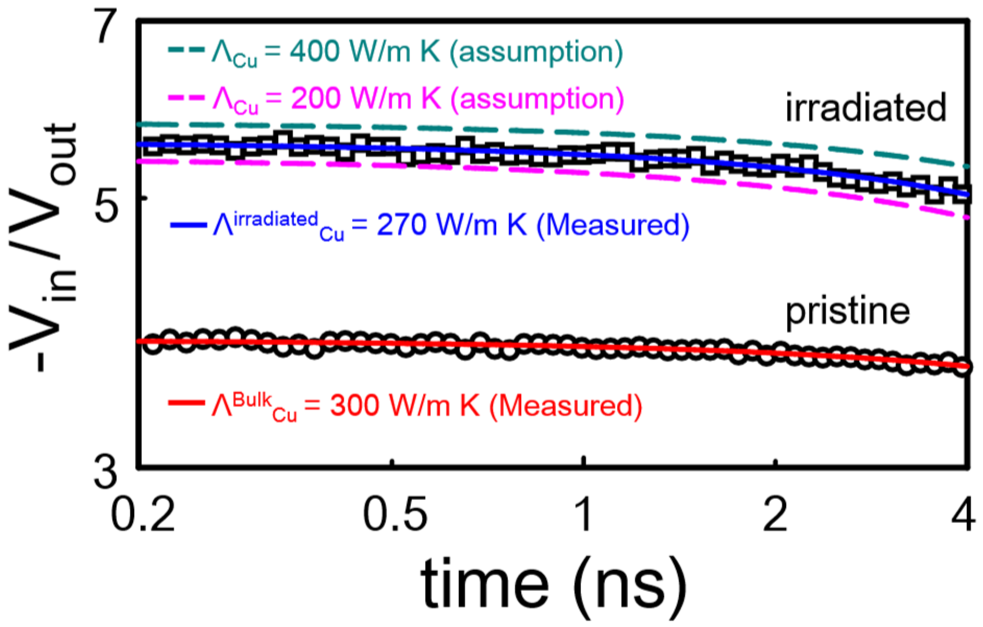

Supplementary Figure S1. Analysis of TDTR data dependent on the change in thermal conductivity of the Cu substrate. The TDTR data and simulation results are plotted with varying thermal conductivity of the Cu substrate. The conductivity Λ_{Cu} changes by ≈10 % due to He^{2+} ion irradiation. The solid lines are the model simulations based on the measured value of the thermal conductivity Λ_{Cu} and the dashed lines are based on assumptions. This plot demonstrates that the TDTR measurement is mostly sensitive to the thermal conductance of the graphene junction, not Λ_{Cu}.

To minimize analytical error, we investigate the Cu substrate effects on the measurement of interfacial thermal conductance G between a metal and graphene. In a metal, the electrical conductivity σ directly converts to thermal conductivity Λ by the Wiedemann–Franz law, $\Lambda/(\sigma T) = L$, where T is the absolute temperature and L is the Lorenz number.[1] While L obtained from the classical free-electron model is $\approx 2.44 \times 10^{-8}$ W Ω K^{-2}, the accurate value depends on

the electronic structure of the material (i.e., band structure and position of the Fermi level). We adopted a value of $L = 2.23 \times 10^{-8}$ W Ω K^{-2} for Cu at room temperature, as given by Kittel.[2] Given this number, we can estimate the thermal conductivity of the poly-crystalline Cu substrate by probing the electrical conductivity. The estimated thermal conductivity (Λ_{cu}) of the Cu substrate is ≈ 300 W m^{-1} K^{-1}; the value of Λ_{Cu} is smaller than the thermal conductivity of single crystal Cu (≈ 400 W m^{-1} K^{-1}). After irradiating with a He^{2+} ion beam, we measure a ≈ 10% reduction in Λ_{Cu} (i.e., ≈ 270 W m^{-1} K^{-1}), which is supposed to be caused by the defect scattering of electrons. The penetration depth of ions at 3.04 MeV is ~ 5.5 μm, which was estimated by the Stopping and Range of Ions in Matter (SRIM) program. The foil thickness is 25 μm. Therefore, we can regard the change in thermal conductivity limited to only a portion of the substrate, but the penetration depth is deep enough for analysis of the TDTR data presented in Supplementary Fig. S1.

S2. Influence of contact area on the thermal transport measurement

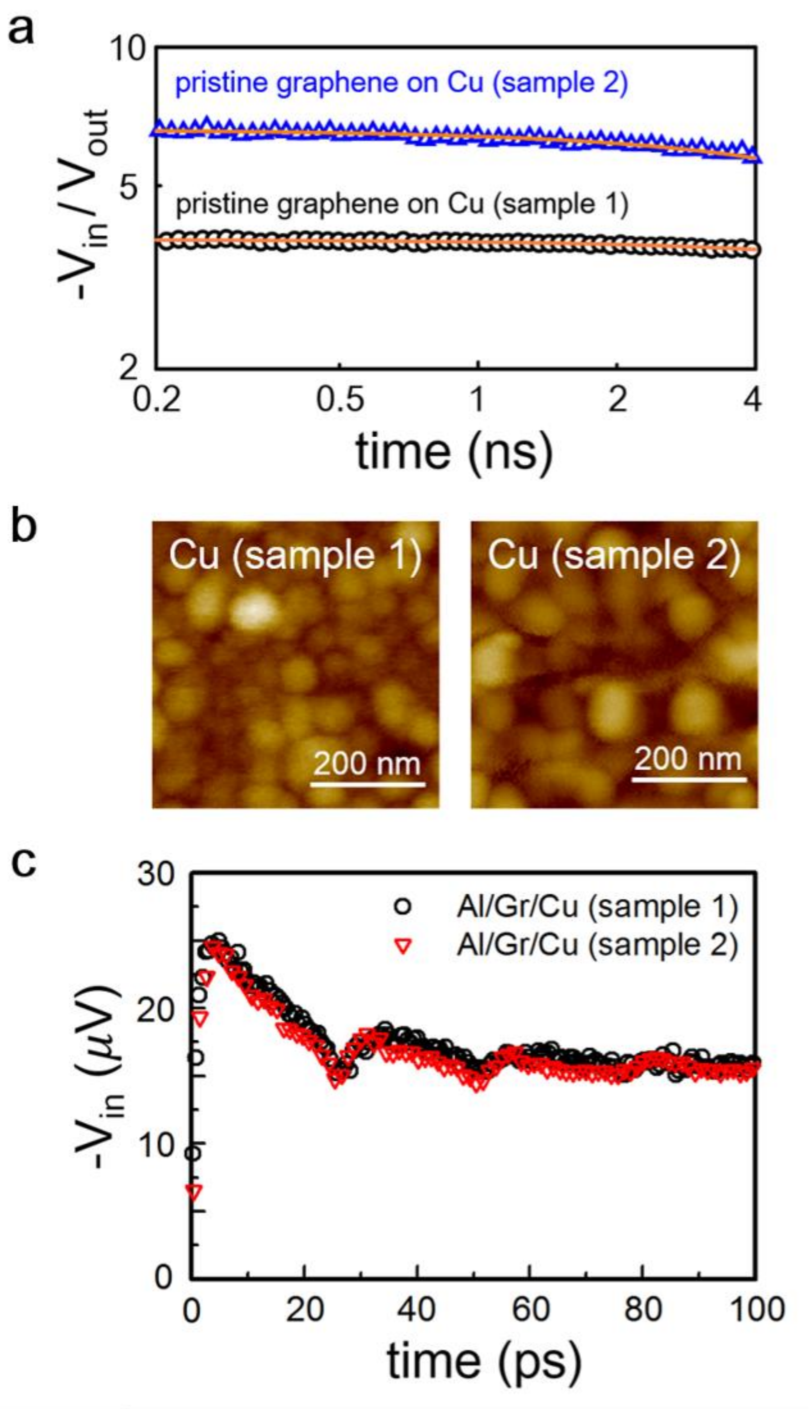

Supplementary Figure S2. (a) TDTR data for sample 2 (blue triangles, $G \approx 31.0$ MW m^{-2} K) and sample 1 grown on different Cu substrates (black circles, $G \approx 21.3$ MW m^{-2} K^{-1}). Solid lines are model calculations fitted to the TDTR data. (b) Atomic force microscope (AFM)

images of graphene grown on two different Cu substrates. (c) In-phase signals (V_{in}) recorded as a function of delay time from the TDTR measurements on both samples.

The measured grain size (D) (root-mean-square roughness) of Cu substrates for sample 1 and sample 2 were 50~70 nm (5 ± 1 nm) and 70~100 nm (3 ± 1 nm), respectively, obtained from the analysis of atomic force microscope (AFM) images as shown in Supplementary Fig. S2b. The Cu grain size of sample 1 is similar to the grain size of Al film, shown in Supplementary Fig. S3, that is later deposited on graphene/Cu substrate, whereas sample 2 has a larger grain size than that of Al film. This difference would lead to a larger contact area for sample 2 and eventually larger thermal conductance than sample 1. On the other hand, this difference hardly affects the signal intensity of the reflected probe beam (i.e., the in-phase signal V_{in}) because of the insignificant difference in the light scattering in heights. In fact, we find that the intensity of the reflected probe beam hardly changed as shown in Supplementary Fig. S2c.

S3. Topographic morphology of the Al thin film

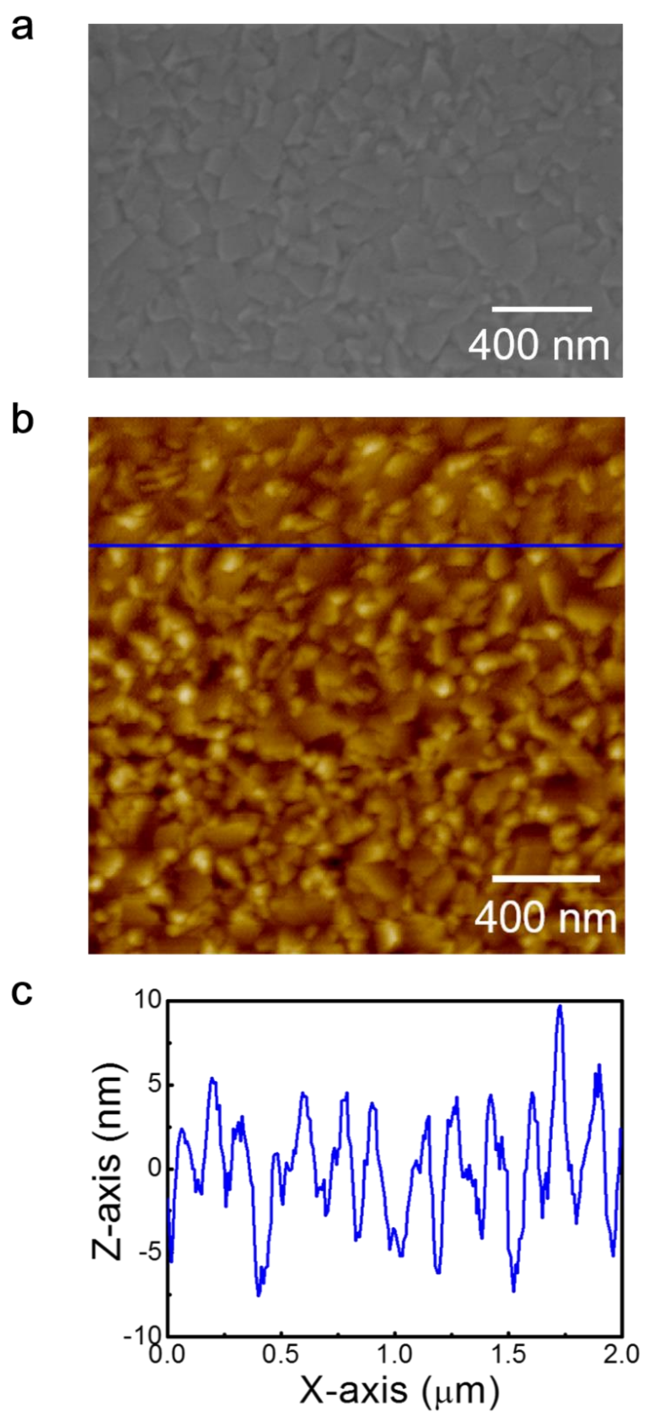

Supplementary Figure S3 (a) SEM image of the surface of the Al thin film for sample 1. (b) Topographic AFM image of the Al thin film. The scan size is 2 μm × 2 μm. (c) Line profile

along the blue solid line in Supplementary Fig. S3b indicates an rms roughness of 5 ± 1 nm. This is similar to the roughness of Cu substrate beneath the film.

In the TDTR measurements, an Al thin film with a thickness of ~80 nm is used as a transducer. We deposit the Al film on the graphene/Cu samples using DC-magnetron sputtering at a base pressure of 5.0×10^{-8} Torr. We analyzed the topographic morphology of the Al film using SEM and an atomic force microscope, as shown in Supplementary Fig. S3. The average size of the grains measured in the Al film is ~ 50 nm. This information also supports that we can exclude the possibility of direct contact between the Al film and the Cu substrate through the ion-irradiated graphene because the average grain size is much larger than the produced defects or even the inter-defect distance in the graphene.

S4. Thermal conductance upon extended exposures to the UV–ozone environment

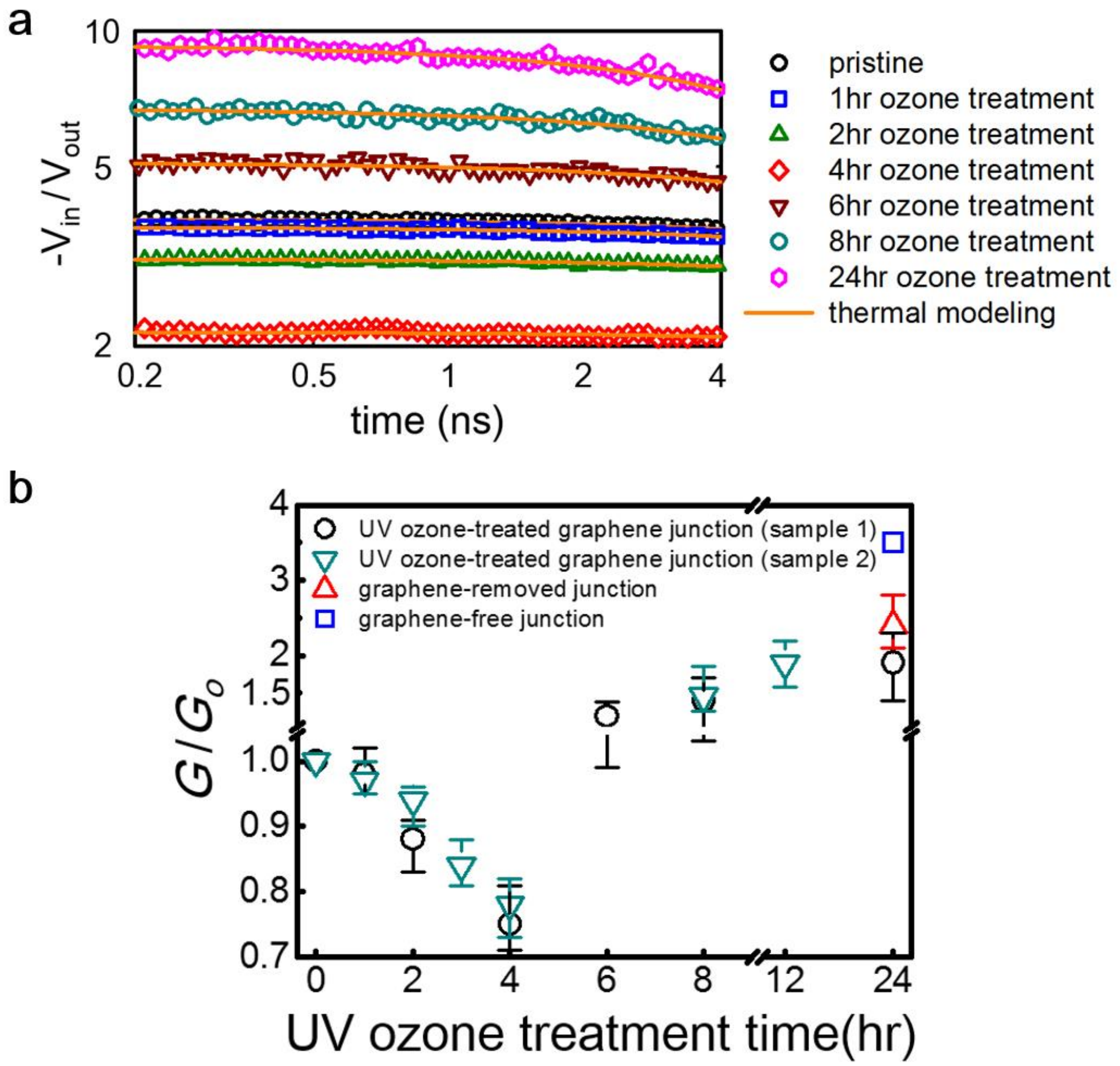

Supplementary Figure S4. (a) TDTR data obtained from pristine, 1-, 2-, 4-, 6- and 24-hr processed graphene samples. The measured conductance values G are ≈ 21.3 MW m^{-2} K^{-1} for the Al/pristine graphene/Cu interface, ≈ 21.2 MW m^{-2} K^{-1} for 1-hr ozone treatment, ≈ 18.8 MW m^{-2} K^{-1} for 2-hr ozone treatment, ≈ 12.9 MW m^{-2} K^{-1} for 4-hr ozone treatment, ≈ 25.0 MW m^{-2} K^{-1} for 6-hr ozone treatment, ≈ 29.5 MW m^{-2} K^{-1} for 8-hr ozone treatment and ≈ 42.0

MW m^{-2} K^{-1} for 24-hr ozone treatment. (b) Thermal conductance change measured from UV-ozone processed graphene for both sample 1 (circle) and sample 2 (reverse-triangle) plotted as a function of exposure time.

The measured conductance decreases roughly linearly with increasing intensity of the Raman D-band, as shown in Fig. 3c, up until four hours of processing. The conductance declines by as much as ~30 % before the conductance increases back up to a saturated value (i.e., as much as twice) at a longer exposure. In Supplementary Fig. S4b, we show all the data obtained from graphene samples grown on Cu substrates with different morphologies as discussed in Supplementary Information S2. The changes in thermal conductance with the oxidation appear quite similar for both samples although the conductance (G_o) for pristine graphene differs by ~50 %. After 24-hour exposure, the conductance value approaches the conductance measured on a sample from which the graphene was removed using oxygen plasma. The large uncertainty after the six-hour treatment is due to spatial non-uniformity. We attribute the non-uniformity and saturating behavior of the thermal conductance to the formation of direct contact between the Al layer and Cu substrate. For comparison, we also show the measured conductance between the Al and Cu substrate by directly depositing an Al layer on Cu substrate. The conductance value is higher than the removed-graphene case where carbon aggregates still remain. However, the value is still ~50 times less than the conductance of the intrinsic[3] Al–Cu interface because of the presence of copper oxide.

S5. Analysis of interfacial thermal conduction using the diffuse mismatch model

In the diffuse mismatch model (DMM), all of the phonons are assumed to be diffusely scattered at the interface and the transmission probability of phonons across the graphene–metal interface can be determined by a mismatch between densities of states on each side. Assuming the density of states of graphite for the graphene, the transmission probability can be written as[4-6]

$$\alpha_{1-2} = \frac{\sum_j \int_0^{\omega_{max}} \hbar\omega v_{2,j} D_2 f_{BE} d\omega}{\sum_j \int_0^{\omega_{max}} \hbar\omega v_{1,j} D_1 f_{BE} d\omega + \sum_j \int_0^{\omega_{max}} \hbar\omega v_{2,j} D_2 f_{BE} d\omega} \ , \qquad (1)$$

where α_{1-2} is the transmission probability from material 1 (i.e., metal) to material 2 (graphite), ω_{max} is the lower cut-off frequency between the materials, D is the phonon density of state (pDOS), v_j is the phonon velocity of mode j, and f_{BE} is the Bose–Einstein distribution. We can then obtain the thermal conductance as

$$G_{1-2} = \frac{1}{4} \sum_j \int_0^{\omega_{max}} \alpha_{1-2} \hbar\omega v_{1,j} D_1 \frac{\partial f_{BE}}{\partial T} d\omega \ . \qquad (2)$$

The cut-off frequency for longitudinal (transverse or flexural) vibrational mode of Al,[7] Cu,[8] and graphene are ~ 9 (5), ~ 8 (5), and ~ 40 (15) THz, respectively. From here, we can calculate the thermal conductance of the interface across the graphene–metal junction. For the oxidized graphene, we obtain a ~ 30% reduction in the conductance with a 40% reduction in the pDOS of graphene.

S6. Computational method: First-principles density functional theory calculation for pristine and oxidized graphene

To compare the vibrational properties of pristine and oxidized graphene, we calculated the pDOS using first-principles density functional theory (DFT) calculations with project-augmented wave (PAW) potentials[9] and the Perdew–Burke–Ernzerhof (PBE) functional[10] as implemented in the Vienna *Ab initio* Simulation Package (VASP).[11] The plane-waves were expanded with a kinetic energy cutoff of 500 eV to obtain basis sets. The atomic structures were optimized to enable more accurate convergence of the calculations until the Hellmann–Feyman forces were less than 10^{-4} eV/A. The pDOS could then be obtained using the small displacement method, as implemented in the VASP package[12] and phonopy program.[13]

S7. Computational method: The non-equilibrium Green's function approach for the interface between the structurally disordered graphene and a metal

To investigate the scientific phenomenon involved in vibrational heat transport through graphene and the metal interface, we used the non-equilibrium Green's function approach combined with the force field method for inter-atomic potentials. The force field approach, implemented in the general utility lattice program (GULP),[14] is utilized to compute the inter-atomic force constants and dynamical matrices. We used the optimized embedded-atom-method (EAM) potential to describe the Cu–Cu[15] and Al–Al[16] interactions, whereas the optimized Tersoff potential is used to explain the C–C interactions.[17] By using this optimized Tersoff potential, we can obtain three acoustic phonon modes that are well-defined at the gamma point in the phonon dispersion of graphene; the frequencies of the vibrational modes in graphene agree well with both the DFT and experimental results.[17] The van der Waals interaction between Al–C and Cu–C atoms at the interface is described by the Lennard–Jones 12-6 potential

$$V_{ij}(\mathrm{r}) = 4\varepsilon_{ij}\left[\left(\frac{\sigma_{ij}}{\mathrm{r}}\right)^{12} - \left(\frac{\sigma_{ij}}{\mathrm{r}}\right)^{6}\right] \quad (2)$$

where ε and σ are Lennard–Jones parameters. The parameters used for calculating the Al–C interaction are ε = 0.038 eV and σ = 2.96 Å,[18] whereas the parameters for the Cu–C interaction are ε = 0.02578 eV and σ = 3.0825 Å.[19]

We compute the phonon transport across the Al/graphene/Cu junction by using the ballistic non-equilibrium Green's function method[20–22] and inter-atomic force constants that can be generated by the force field approach along with an-harmonic approximation. Retarded Green's function for the channel or device region can be calculated as

$$G(\omega) = [(\omega + i\eta)^2 I - K_C - \Sigma_L - \Sigma_R]^{-1} \quad (3)$$

where ω is the vibration frequency of phonons, I is the identity matrix, and K_C is the dynamical matrix calculated for the central device or channel region, which is the sandwiched graphene layer with defects. $\Sigma_L = K^{\dagger}_{LC}\, g_L\, K_{LC}$ and $\Sigma_R = K_{CR}\, g_R\, K^{\dagger}_{CR}$ are self-energies due to semi-infinite left and right leads, where g_L and g_R are the surface Green's functions that can be calculated through a recursive iterations method.[23] We obtain the phonon transmission coefficients $\overline{T}(\omega)$ by

$$\overline{T}(\omega) = \mathrm{Tr}[\Gamma_L(\omega) G(\omega)\, \Gamma_R(\omega) G^{\dagger}(\omega)] \quad (4)$$

where $\Gamma_{L(R)} = i[\Sigma_{L(R)} - \Sigma^{\dagger}_{L(R)}]$ is the broadening function of the left (right) lead and $G^{\dagger}$ is the advanced Green's function. Thermal conductance can then be computed using the Landauer formula[24–30]

$$\sigma(T) = \int_0^{\infty} \frac{d\omega}{2\pi} \hbar\omega \overline{T}(\omega) \frac{\partial f_{BE}}{\partial T} \quad (5)$$

where f_{BE} is the Bose–Einstein distribution function for phonon occupation at the leads.

The atomic structure of the computational model is shown in Fig. 4b, in which a 15 × 15 graphene sheet is sandwiched between 14 × 14 aluminum (111) and 16 × 16 copper (111) substrate. We used the (111) surface; in this combination, 14 × 14 Al and 16 × 16 Cu have a 1.7% lattice mismatch with the 15 × 15 graphene sheet. The lattice constant of graphene is utilized for the supercell structure consisting of Al/Gr/Cu. The disorder is introduced in the form of mono-atomic vacancies in the graphene layer. Figure 4c depicts the graphene layers incorporated with 1 and 3% defect densities in its left and right panels, respectively. To calculate the atomistic Green's function, single-layer graphene is considered a device that is sandwiched between Al and Cu that behaves as left and right leads or contacts.

S8. Flexural pDOS analysis to elucidate increasing thermal conduction with graphene/metal vibrational coupling

To understand the modified coupling at the metal–graphene interface, the phonon modes that participate in interfacial heat transport are comprehensively analyzed. We find that the out-of-plane phonon modes are major entities that play a key role in the thermal transport between the graphene and the metal. Therefore, coupling between the graphene and the metal can be understood more accurately through intimate analysis of the projection of phonon eigenvectors along the out-of-plane direction of the graphene layer. The out-of-plane pDOS for the pristine and defective sandwiched graphene are presented in Fig. 4b. Since we are mainly interested in the frequency range of 0–8 THz, only the lower frequency range is included in the plot. This frequency cutoff is decided by the lowest frequency among the three materials involved, which is about 8 THz for Cu.[8]

The flexural pDOS in Fig. 4b reveals an increase in the acoustic out-of-plane states after vacancy incorporation. This evidently indicates that the increased out-of-plane phonon coupling strength between the graphene and metal contacts results from defects in the graphene layer. Further characterization of pDOS at Al/Gr/Cu interfaces with vacancies in the graphene layer has exhibited several out-of-plane phonon modes below 4.6 THz that originated from vacancy-type disorder. Additionally, a slight increase in the in-plane phonon states is observed below 8 THz, whereas a reduction in both the in-plane and out-of-plane modes—because of defects—happens over 8 THz. Although the in-plane modes are mixed with out-of-plane modes below 8 THz, those modes are not directly involved in cross-plane phonon transmission.

Animations of atomic oscillations are presented in Supplementary Video SV1 for the frequency range below 8 THz of the phonon modes related to the enhanced peak in the projected out-of-plane density of states for the sandwiched graphene layer with 3 % defect

density (see Fig. 4b). The arrows along the atoms in the animation video indicate the magnitude of the vibrational forces and their oscillation direction. The incorporation of vacancies has broken the symmetry of the sandwiched graphene. This drives the atoms neighboring the vacancies to behave as unconnected springs. It is found that oscillations from 4 to 5.6 THz mainly develop around the vacancies, and that all of these are vertical vibrations that induce a remarkable increase in the flexural pDOS. Therefore more heat can flow through the graphene–metal interface because of enhanced coupling. Mixing between the in-plane and out-of-plane modes is also observed along with an increase in vertical oscillation around the defects; this type of vibrational behavior is also included in Supplementary Video SV1 for a few phonon modes between 5.6 and 6.5 THz. In contrast, a further increase in the density of vacancies over 3 % distorts the graphene layer and the coupling strength at the metal–graphene interface becomes weakened, which results in the reduction of heat transport from vacancy densities of 3 % and up; this is visualized in the interfacial thermal conduction plot in Fig. 4c.

Within the frame, the phonon transmission probability across the Al/Gr/Cu junction was calculated, as shown in Supplementary Fig. S5. The calculation requires the pDOS projected on the out-of-plane and in-plane modes, which are shown in Fig. 4b. Note that the out-of-plane mode at Γ is lifted from 0 to ~1 THz due to the Cu substrate and Al film. As we showed in Supplementary Fig. S5, the phonon transmission of the in-plane modes is essentially negligible while the out-of-plane modes are dominant over the frequency range of interest (below 8 THz).

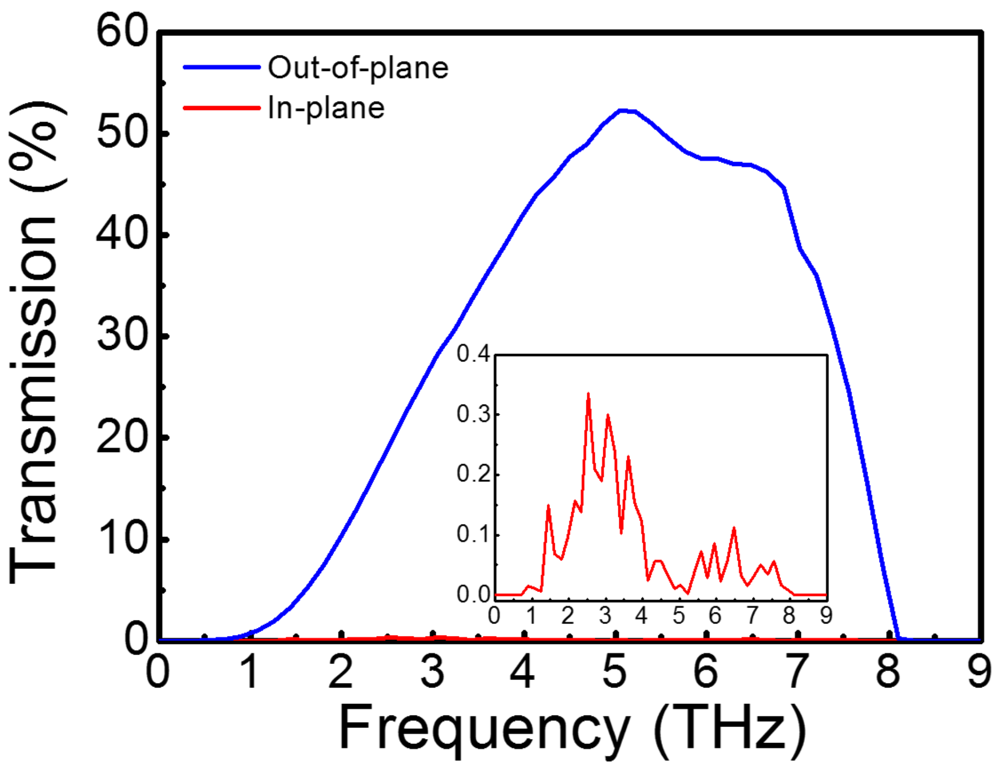

Supplementary Figure S5. Phonon transmission probability across the Al/Gr/Cu junction. Calculated transmission probability of phonon modes was plotted along each projection (i.e., blue line: out-of-plane phonon modes, red line: in-plane phonon modes), where the inset exhibits a close-up of the in-plane modes. Despite the shift of pDOS (Fig. 4b) due to the substrate, the out-of-plane modes dominate the transmission.

References

1. Jones, W. & March, N. H. *Theoretical solid state physics: Perfect lattices in equilibrium*. Vol. 1 (Courier Corporation, 1973).
2. Kittel, C. *Introduction to solid state physics*. (Wiley, 2005).
3. Gundrum, B. C., Cahill, D. G. & Averback, R. S. Thermal conductance of metal-metal interfaces. *Phys. Rev. B* **72**, 245426 (2005).
4. Swartz, E. T. & Pohl, R. O. Thermal boundary resistance. *Reviews of modern physics* **61**, 605 (1989).
5. Duda, J. C., Smoyer, J. L., Norris, P. M. & Hopkins, P. E. Extension of the diffuse mismatch model for thermal boundary conductance between isotropic and anisotropic materials. *Applied Physics Letters* **95**, 031912 (2009).
6. Schmidt, A. J., Collins, K. C., Minnich, A. J. & Chen, G. Thermal conductance and phonon transmissivity of metal–graphite interfaces. *J. Appl. Phys.* **107**, 104907 (2010).
7. Gupta, H. & Tripathi, B. Pseudopotential and the Phonon Dispersion in Aluminum. *Phys. Rev. B* **2**, 248 (1970).
8. Dal Corso, A. Ab initio phonon dispersions of transition and noble metals: effects of the exchange and correlation functional. *Journal of Physics: Condensed Matter* **25**, 145401 (2013).
9. Blöchl, P. E. Projector augmented-wave method. *Phys. Rev. B* **50**, 17953 (1994).
10. Perdew, J. P., Burke, K. & Ernzerhof, M. Generalized gradient approximation made simple. *Physical review letters* **77**, 3865 (1996).
11. Kresse, G. & Joubert, D. From ultrasoft pseudopotentials to the projector augmented-wave method. *Phys. Rev. B* **59**, 1758 (1999).
12. Parlinski, K., Li, Z. & Kawazoe, Y. First-principles determination of the soft mode in cubic ZrO_2. *Physical Review Letters* **78**, 4063 (1997).
13. Togo, A., Oba, F. & Tanaka, I. First-principles calculations of the ferroelastic transition between rutile-type and $CaCl_2$-type SiO_2 at high pressures. *Phys. Rev. B* **78**, 134106 (2008).
14. Gale, J. D. & Rohl, A. L. The General Utility Lattice Program (GULP). *Molecular Simulation* **29**, 291–341, (2003).
15. Johnson, R. A. Alloy models with the embedded-atom method. *Phys. Rev. B* **39**, 12554–12559 (1989).
16. Mei, J. & Davenport, J. W. Free-energy calculations and the melting point of Al. *Phys. Rev. B* **46**, 21–25 (1992).
17. Lindsay, L. & Broido, D. A. Optimized Tersoff and Brenner empirical potential parameters for lattice dynamics and phonon thermal transport in carbon nanotubes and graphene. *Phys. Rev. B* **81**, 205441 (2010).
18. Xiao, S. & Hou, W. Fracture of vacancy-defected carbon nanotubes and their embedded nanocomposites. *Phys. Rev. B* **73**, 115406 (2006).
19. Xu, Z. & Buehler, M. J. Nanoengineering Heat Transfer Performance at Carbon Nanotube Interfaces. *ACS Nano* **3**, 2767–2775, (2009).
20. Mingo, N. Anharmonic phonon flow through molecular-sized junctions. *Phys. Rev. B* **74**, 125402 (2006).
21. Wang, J.-S., Wang, J. & Zeng, N. Nonequilibrium Green's function approach to mesoscopic thermal transport. *Phys. Rev. B* **74**, 033408 (2006).
22. Wang, J.-S., Zeng, N., Wang, J. & Gan, C. K. Nonequilibrium Green's function method for thermal transport in junctions. *Physical Review E* **75**, 061128 (2007).

23. Sancho, M. P. L., Sancho, J. M. L. & Rubio, J. Highly convergent schemes for the calculation of bulk and surface green-functions *Journal of Physics F-Metal Physics* **15**, 851–858, (1985).
24. Dubi, Y. & Di Ventra, M. Heat flow and thermoelectricity in atomic and molecular junctions. *Rev. Mod. Phys.* **83**, 131–155 (2011).
25. Jeong, C., Kim, R., Luisier, M., Datta, S. & Lundstrom, M. On Landauer versus Boltzmann and full band versus effective mass evaluation of thermoelectric transport coefficients. *J. Appl. Phys.* **107**, 023707 (2010).
26. Markussen, T., Jauho, A.-P. & Brandbyge, M. Electron and phonon transport in silicon nanowires: Atomistic approach to thermoelectric properties. *Phys. Rev. B* **79**, 035415 (2009).
27. Reddy, P., Jang, S.-Y., Segalman, R. A. & Majumdar, A. Thermoelectricity in Molecular Junctions. *Science* **315**, 1568–1571, 1137149 (2007).
28. Sivan, U. & Imry, Y. Multichannel Landauer formula for thermoelectric transport with application to thermopower near the mobility edge. *Phys. Rev. B* **33**, 551–558 (1986).
29. Widawsky, J. R., Darancet, P., Neaton, J. B. & Venkataraman, L. Simultaneous Determination of Conductance and Thermopower of Single Molecule Junctions. *Nano Lett.* **12**, 354–358, (2011).
30. Yang, K. *et al.* Enhanced thermoelectric properties in hybrid graphene/boron nitride nanoribbons. *Phys. Rev. B* **86**, 045425 (2012)
